# Modulating the catalytic activity of AMPK has neuroprotective effects against α-synuclein toxicity

**DOI:** 10.1186/s13024-017-0220-x

**Published:** 2017-11-03

**Authors:** Wojciech Bobela, Sameer Nazeeruddin, Graham Knott, Patrick Aebischer, Bernard L. Schneider

**Affiliations:** 1 Brain Mind Institute, Ecole Polytechnique Fédérale de Lausanne (EPFL), Station 19, 1015 Lausanne, Switzerland; 2 Centre of Interdisciplinary Electron Microscopy, Ecole Polytechnique Fédérale de Lausanne (EPFL), 1015 Lausanne, Switzerland

**Keywords:** Aging, AMPK, α-synuclein, Neuroprotection, Parkinson’s disease

## Abstract

**Background:**

Metabolic perturbations and slower renewal of cellular components associated with aging increase the risk of Parkinson’s disease (PD). Declining activity of AMPK, a critical cellular energy sensor, may therefore contribute to neurodegeneration.

**Methods:**

Here, we overexpress various genetic variants of the catalytic AMPKα subunit to determine how AMPK activity affects the survival and function of neurons overexpressing human α-synuclein in vivo.

**Results:**

Both AMPKα1 and α2 subunits have neuroprotective effects against human α-synuclein toxicity in nigral dopaminergic neurons. Remarkably, a modified variant of AMPKα1 (T172Dα1) with constitutive low activity most effectively prevents the loss of dopamine neurons, as well as the motor impairments caused by α-synuclein accumulation. In the striatum, T172Dα1 decreases the formation of dystrophic axons, which contain aggregated α-synuclein. In primary cortical neurons, overexpression of human α-synuclein perturbs mitochondrial and lysosomal activities. Co-expressing AMPKα with α-synuclein induces compensatory changes, which limit the accumulation of lysosomal material and increase the mitochondrial mass.

**Conclusions:**

Together, these results indicate that modulating AMPK activity can mitigate α-synuclein toxicity in nigral dopamine neurons, which may have implications for the development of neuroprotective treatments against PD.

**Electronic supplementary material:**

The online version of this article (10.1186/s13024-017-0220-x) contains supplementary material, which is available to authorized users.

## Background

Parkinson’s disease (PD) is a debilitating neurodegenerative disease mainly characterized by motor symptoms, which result from a dysfunction of basal ganglia caused by degeneration of dopamine neurons in the substantia nigra pars compacta (SNpc). Alpha-synuclein (α-syn), a small presynaptic protein highly expressed in the dopamine neurons of the ventral midbrain, plays an important role in PD etiology [1, 2], via mechanisms involving its misfolding and aggregation [3, 4].

Apart from rare genetic forms with early onset [5], incidence of the disease depends on age and therefore, aging is considered a major risk factor for PD. The exact nature of the interplay between aging and pathogenic mechanisms remains however poorly explored. Nevertheless, it has been shown that α-syn protein levels increase significantly with age [6]. Aging may also impair capacity of cells to cope with metabolic stress. This may affect the survival and function of neurons exposed to high energy demand, such as nigral dopamine neurons [7]. Decreased turnover rates of organelles and proteins, including α-syn, may further contribute to metabolic defects and prompt neurodegeneration.

AMP-activated protein kinase (AMPK) is a molecular gauge of energy status, at both cellular and whole-body levels [8, 9]. Mammalian AMPK is a heterotrimeric complex consisting of α, β and γ subunits, which carry out enzymatic, scaffolding and regulatory functions, respectively [8–11]. In mammals, there exist two isoforms of the α and β subunits, and three isoforms of the γ subunit, all encoded by separate genes [8, 9, 12]. AMPK becomes catalytically active when cellular levels of AMP rise substantially, as a consequence of metabolic stress. Binding of AMP to the CBS domains in γ subunit, triggers allosteric activation of the complex, favors T172 phosphorylation by AMPK kinases, and protects this residue from potential dephosphorylation. As a consequence of AMP binding, the catalytic activity of the AMPK complex is increased by nearly 1000-fold [8, 9, 13–15]. Once activated, AMPK induces catabolic processes and inhibits energy consumption, in order to maintain metabolic homeostasis [8, 9, 11, 16].

A growing body of evidence indicates a role for AMPK in aging, which might possibly be linked to the risk of developing neurodegenerative diseases, including PD. AMPK signaling gradually declines with age [17–20], whilst activation of AMPK and its downstream targets has been shown to increase longevity in model organisms like Drosophila [21] and *C. elegans* [22]. Remarkably, low activity of peroxisome proliferator-activated receptor γ coactivator-1α (PGC-1α), one of the key downstream effectors of AMPK, has been linked to sporadic PD cases [23]. Furthermore, metformin and glitazone, anti-diabetic drugs acting via AMPK and PGC-1α, respectively, have been shown to significantly decrease the risk of PD in large cohort clinical trials [24, 25].

Only a few studies have explored the effect of AMPK on α-syn toxicity. In vitro, overexpression of α-syn lowers AMPK activity, which can be compensated by exposure to metformin or 5-aminoimidazole-4-carboxamide-1-β-D-ribofuranoside (AICAR). Conversely, reduced expression of AMPK lowers cellular resistance to α-syn [26]. However, inducing chronic high AMPK activity was also found to be detrimental in cell-based assays [27]. It is well recognized that α-syn may also perturb key cellular processes including mitochondrial activity, which may indirectly impinge on AMPK signaling. Recently, α-syn has been found to bind PIKE-L, an inhibitor of AMPK, as a function of S129 phosphorylation [28]. PIKE sequestration in Lewy bodies may hence contribute to neuronal degeneration via AMPK overactivation. Overall, it is however not known how AMPK may control the survival of nigral dopamine neurons exposed to the α-syn pathology in vivo.

Here, we explore with a genetic approach how the activity of the AMPK complex determines vulnerability of neurons overexpressing α-syn. In order to modulate the energy-sensing ability of the complex, we overexpress variants of the AMPKα subunit, which differ in their activity pattern, and test their neuroprotective effects in neurons overexpressing human α-syn. In vivo, our results provide evidence that the catalytic AMPKα subunit is neuroprotective at early stages of the developing α-syn pathology. In particular, the AMPK T172Dα1 variant, which provides mild chronic catalytic activity, leads to the most efficient neuroprotection by reducing the accumulation of misfolded α-syn within dopamine axons projecting to the striatum (STR). In vitro, we show that the overexpression of AMPKα subunits in primary neurons, modifies the effects of α-syn on autophagic and mitochondrial activities. These results suggest that pre-emptive measures to chronically activate AMPK or enhance the catalytic response of this metabolic sensor, can mitigate the effects of α-syn toxicity in a genetic rodent model of PD pathology.

## Methods

### Vector construction

Plasmid constructs encoding the K45Rα2 and 1-310α2 variants were generated from human wild-type AMPKα2 (nucleotides 72–1730, NM_006252) via site-directed mutagenesis and PCR amplification. These sequences, as well as the α-syn cDNA (nucleotides 46–520, NM_000345), were introduced into the pAAV-pgk-MCS-WPRE backbone using standard cloning procedures. Similar pAAV-pgk vector constructs encoding wild-type human AMPKα1 and the T172Dα1 variant were kindly provided by Dr. K. Sakamoto. To assess construct functionality, each of the pAAV-AMPKα plasmid was co-transfected in HEK293T cells, either alone or in combination with plasmids encoding the β1 (pECE-HA-AMPKbeta1, Addgene #31666) and γ3 subunits of AMPK (pDONR223-PRKAG3, Addgene #23549). The pAAV constructs encoding the mCherry-GFP-LC3 reporter of autophagy and mitoDsRed have been previously described [29, 30].

### Production of AAV2/6 vector particles and vector titration

Viral vectors were produced and titrated as previously described [31]. Briefly, relative AAV infectivity was determined by real-time PCR (rtPCR) quantification of double-stranded vector genomes present in total DNA isolated from HEK293 cells, 48 h post-infection. The infectivity rate expressed in ‘transducing units’ (TU) was calculated according to a known infectivity of a standard virus encoding GFP (AAV2/6-cmv-eGFP), whose titer was estimated via flow cytometry.

### Cultures of mouse primary cortical neurons

Primary cortical neurons were derived from C57BL6/J mouse embryos, at day E16.5. Unless stated otherwise, cells were cultured in the Neurobasal medium (Thermofisher Scientific #21103–049), supplemented with 2% B27 (Thermofisher Scientific #17504–044), 1% GlutaMax (Thermofisher Scientific #35050–061) and 1% mix of Penicillin/Streptomycin (Thermofisher Scientific #10378–016) at 37 °C and 5% CO_2_. For all biochemical purposes, neurons were grown on adequate culture plates, pre-coated overnight at 37 °C, with 100 μg/ml poly-DL-ornithine (Sigma #P8638). For immunocytochemistry assays, neurons were grown in the 24-well plate formats, on glass cover slips pre-coated with 0.2 mg/ml poly-D-lysine (Sigma #P6407) and 33.2 μg/ml laminin (Thermofisher Scientific #23017015).

### Mitochondrial DNA and mitochondrial mass estimation

Neurons were plated at a density of 200,000 per well in a 24-well plate format, in medium without phenol red (Thermofisher Scientific #12348–017). On the fifth day, they were infected with AAV2/6-mitoDsRed vector in combination with the other vectors mentioned in the text. All vectors were used at a dose of 0.8E6 TU. Seven days later, neurons were collected by gentle dissociation. The arithmetic mean of mitoDsRed red fluorescence intensity was determined using Accuri C6 Flow Cytometer, recording events in the gated population of living cells.

To estimate the amount of mitochondrial DNA (mtDNA) using rtPCR, primary neurons were infected with AAV2/6 vectors at a dose of 1E6 TU for each vector on day 5 after plating. On day 12, cells were lysed and total DNA was extracted, following Maxwell 16 Viral Total Nucleic Acid Purification Kit protocol (Promega). SYBR-green rtPCR (Qiagen) was used to determine the relative amounts of mtDNA and gDNA in each sample, using two sets of primers: 16S rRNA for mtDNA and Hexokinase-2 (Hk2) for gDNA. The relative amounts of mtDNA/gDNA were determined using the ΔΔCt method. For the primer sequences please refer to the Supplementary Methods (Additional file 1).

### Estimation of the autophagic activity using LC3B-mCherry-EGFP reporter

Neurons were plated at a density of 200,000 cells on glass cover slips, pre-coated with laminin and poly-D-lysine, and cultured in medium without phenol red. On day 5 after plating, neurons were infected with 1E6 TU of each AAV2/6 vector. On day 12, cultures were fixed for 20 min with ice-cold 4% paraformaldehyde (PFA). Following staining with DAPI, coverslips were mounted on glass slides using Mowiol medium (Fluka).

Neurons expressing the AAV-encoded LC3B-mCherry-EGFP probe were analyzed using Zeiss LSM 700 confocal microscope at 63× magnification. Pictures were taken in random fields. Each condition was run in triplicates, for a total number of 22–30 neurons per condition. Yellow or red dots, corresponding to autophagosomes and autolysosomes, respectively, were counted manually, in a blind manner, after subtracting mean values of fluorescent signal in each channel. Numbers of autophagic vesicles were normalized to cytosol area. The analysis was performed using Fiji software plugins.

### Biochemical analysis of protein expression

Neurons were plated at a density of 650,000 per well in 12-well plate format. On day 5 after plating, neurons were infected with 3.9E7 TU of each AAV2/6 vector. At day 12, neurons were harvested in lysis buffer containing 0.5% NP40 with protease and phosphatase inhibitors (Roche), and incubated on ice for 10 min. Following cell lysis, protein extracts were centrifuged for 20 min at 14,000 rpm at 4 °C and the supernatant was collected. Protein concentration was evaluated using BCA Protein Kit Assay (Thermofisher Scientific #23225). For detailed description of the protein analysis, refer to Supplementary Methods (Additional file 1).

Primary antibodies: AMPK alpha (Cell Signaling #2532) 1:1000; pAMPK alpha (Thr 172) (40H9; Cell Signaling #2535) 1:1000; pACC (Millipore #07–303) 1:1000; Actin (I-19; Santa Cruz #sc-1616) 1:5000; anti-α-syn (Becton Dickinson Biosciences #610787) 1:8000; anti-MAP1LC3B (Lifespan BioSciences) 1:1000. Secondary antibodies: Goat Anti-Rabbit IgG H&L Chain Specific Peroxidase Conjugate (Calbiochem #401353), Goat Anti-Mouse IgG H&L Chain Specific Peroxidase Conjugate (Calbiochem #401215).

### Stereotaxic injection of viral vectors

All in vivo experiments were performed using adult female Sprague-Dawley rats (Janvier), weighing around 200 g at the time of surgical procedure. Animals were housed in standard 12 h light/dark cycles, with ad libitum access to water and food. All procedures were approved by a local ethics committee and performed in accordance with the Swiss legislation and the European Community Council directive (86/609/EEC) regulating care and use of laboratory animals. In order to minimize stress, animals were accustomed for at least one week prior to behavioral test and surgical manipulation. For detailed description of the injection procedure, refer to Supplementary Methods (Additional file 1).

Coordinates used to target the SNpc: −5.2 mm (anteroposterior), −2 mm (mediolateral), −7.8 mm (dorsoventral, relative to skull surface), −3.3 mm (tooth bar). Vectors were used at a total injected dose of 1.5E7 TU (AAV2/6-α-syn) and 1.2E6 TU (AAV2/6-AMPKα and AAV2/6-non-coding vector).

### Animal behavior

During the time course of the study, spontaneous forelimb activity was estimated periodically using the cylinder test. Animal’s performance was evaluated during 5 min. The time was extended in case an animal was poorly active, until it cumulatively used both of its forepaws a minimum of 20 times.

Right forepaw preference was calculated according to the following formula:

$$ \mathrm{RPx}\left(\%\right)=\left(\frac{\mathrm{Rx}}{\mathrm{Rx}+\mathrm{Lx}}-\frac{\mathrm{Ro}}{\mathrm{Ro}+\mathrm{Lo}}\right)\times 100 $$

Where: RP_x_-right forepaw preference at a given time point; R_x_-total number of the right forepaw use at a given time point; R_0_- total number of the right forepaw use before surgery; L_x_- total number of the left forepaw use at a given time point; L_0_- total number of the left forepaw use before surgery.

### Immunohistochemistry

Procedures for the preparation of brain tissues and immunohistochemistry are described in the Supplementary Methods (Additional file 1).

Primary antibodies (immunofluorescence): anti-TH (Millipore #AB152) 1:1000; anti-α-syn (Millipore #AB5334P) 1:1000; anti-α-syn clone 5G4 (AJ Roboscreen) 1:1000; anti-phospho S129 α-syn (Abcam ab59264) 1:1000. Secondary antibodies: Cy2-conjugated donkey anti-rabbit IgG (H + L) (Jackson ImmunoResearch Inc. #711–225-152) 1:1000; Cy3-conjugated F(ab’)_2_ fragment donkey anti-sheep IgG (H + L) (Jackson ImmunoResearch Inc. #713–166-147) 1:1000.

Primary antibodies (DAB): anti-TH (Millipore #AB152 1:1000); anti-DAT (Millipore #MAB369) 1:4000; anti-HA-tag (Covance clone 16B12 #MMS-101P) 1:1000. Secondary antibodies: peroxidase Goat anti-rabbit IgG (Vector Laboratories #PI-1000) 1:200; peroxidase goat anti-mouse IgG (Vector Laboratories #BA-9200) 1:200; biotinylated rabbit anti-rat IgG (Vector Laboratories #BA-4001).

### Optical densitometry and stereological evaluation of neuron loss

On average, a total number of 16 sections (one in six interval) covering the whole STR were DAB-stained for tyrosine hydroxylase (TH) or dopamine transporter (DAT) in order to visualize dopamine fibers. Stained sections were scanned using Epson Perfection V750 Pro scanner. For each STR section, optical density, defined as integrated density of grey pixel values corrected for background noise and striatal surface, was measured in each hemisphere using the ImageJ software. For each animal, results are expressed as a percent loss of total optical density on the injected, compared to non-injected side.

Total α-syn overexpression in the midbrain was evaluated by fluorescence immunohistochemistry (anti-α-syn, Millipore #AB5334P). Three sections near the site of vector injection were imaged using a slide scanner (Olympus VS120-L100). Integrated fluorescence intensity was determined on three sections in the entire midbrain of the right hemisphere using Fiji software, after subtraction of the background intensity measured on the same sections in the contralateral hemisphere.

Aggregated forms of α-syn were visualized in the rat STR using immunostaining with 5G4 anti-α-syn monoclonal antibody. For the analysis, we used 5–6 sections per animal. Fluorescent pictures of the injected side were taken in the dorsal part of medial STR using a Leica DM5500 microscope, and assembled into a mosaic image. Optical densitometry of a given area was calculated using the ImageJ software. Data represent the mean ± SEM of the grey value of pixels, averaged from 5 to 6 sections of the medial STR per animal.

Detailed description of the procedures for stereological assessment of neuron counts are provided in the Supplementary Methods (Additional file 1).

### Transmission electron microscopy

For detailed description of sample preparation, refer to Supplementary Methods (Additional file 1). Each condition consisted of neurons analyzed from two animals. Thin sections in the SNpc region were viewed in the electron microscope and images taken of every neuronal cell body containing profiles of nuclei. Only large neuronal cell bodies were imaged. All the mitochondria and the cytosol were annotated in the TrakEM2 software running in the Fiji software. Relative comparisons of mitochondrial size, density, and their volume fraction in the cytosol were based on the 2D images.

### Statistical analysis

All data are expressed as arithmetic mean with a standard error of the mean (SEM). If not mentioned otherwise, statistical analysis of the data was performed using one- or two-way analysis of variance (ANOVA), with a subsequent Tukey’s honest significant difference (HSD) post hoc test, using Statistica software (Statsoft). The alpha level of significance was set at 0.05. For each experiment, the number of replicates is indicated in the figure legend. The experimenter acquiring data was blinded to the experimental protocol.

## Results

### AAV-based constructs for overexpression of AMPKα modulate AMPK activity in neuronal cells

**Fig. 1:**
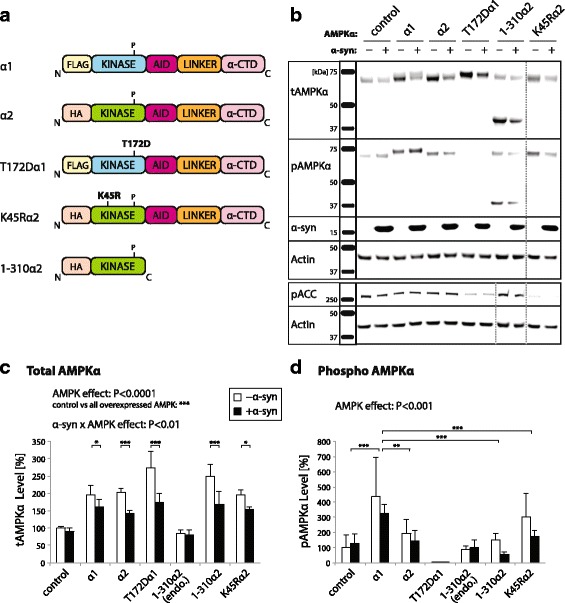
Co-expression of AMPKα subunits and α-syn in mouse cortical neurons. **a** AMPKα constructs used in the study, overexpressed using AAV2/6 vectors: wild-type human α1 and α2 forms; T172Dα1 mutant mimicking constitutive phosphorylation of threonine 172; K45Rα2 kinase dead mutant and 1-310α2 truncated form lacking auto-inhibitory loop and ability to integrate in the AMPK complex. **b** Primary cortical neurons transduced with α-syn-encoding vector (or non-coding vector as control) were co-transduced with AMPKα expressing vectors. Protein analysis by western blotting shows total AMPK (tAMPKα), T172-phosphorylated AMPKα (pAMPKα), α-syn, S79-phosphorylated ACC (pACC) and actin. Analysis is performed at day 7 after infection. **c** Relative quantification of tAMPK levels normalized to actin. Transduction with AAV-AMPKα increases tAMPK levels. Note that α-syn overexpression leads to a significant decrease in tAMPK protein levels. **d** Relative quantification of pAMPK level normalized to actin. Note that the overexpression of α-syn does not induce any significant change in T172 phosphorylation and that overexpressed α1 is more efficiently phosphorylated than other AMPKα variants. *Statistical analysis*: repeated measures two-way ANOVA with Fisher‘s LSD *post hoc* test; n=3 per condition; *P<0.05; **P<0.01; ***P<0.001. In panels c, d: ‘1-310α2 endo’ refers to the level of endogenous tAMPK and pAMPK (upper bands) in neurons overexpressing the 1-310α2 variant

We generated AAV vector constructs encoding different variants of AMPKα to modulate activity of the AMPK complex. These constructs are described in Fig. 1a. They comprise wild-type forms of the AMPKα1 and α2 subunits, as well as the catalytically inactive K45R mutant of the α2 subunit (K45Rα2). In addition, two previously characterized constitutively active forms of AMPKα were included in our experiments: T172Dα1 encodes a full-length AMPKα1 mutant, which carries an aspartate residue on the critical position 172 (T172D) to mimic phosphorylation. This variant has low but constitutive activity [32, 33]; 1-310α2 encodes a constitutively active truncated version of AMPKα2 (amino acids 1–310), which does not integrate into the AMPK complex because it is devoid of both the C-terminal auto-inhibitory domain (AID) and the β subunit-binding domain (α-CTD) [34]. To assess the activity of these constructs as a function of the amount of AMPK complex, we used an assay based on AMPK overexpression in HEK293T cells and measured the level of phospho-acetyl-CoA carboxylase (pACC), a product of AMPK activity (Additional file 2: Fig. S1). To determine if the activity of each of these variants was dependent on the availability of other AMPK subunits, we compared a condition in which only the α subunit was overexpressed, with a condition in which the β1 and γ3 subunits were co-overexpressed with the α subunit. In cells expressing either the AMPKα1 or the α2 subunit, pACC levels appeared to increase when the β and γ subunits were co-overexpressed (Additional file 2: Fig. S1), indicating that AMPK activity is controlled by complex formation. However, in cells overexpressing the 1-310α2, T172Dα1 and K45Rα2 subunits, the level of pACC remained very similar in both conditions, which shows that the activity of these variants is to a large extent independent from the AMPK complex. Furthermore, the level of pACC was found to be higher with the 1-310α2 subunit, as compared to the T172Dα1 and K45Rα2 subunits.

To assess the effects of AMPKα overexpression in neuronal cells, we next transduced primary neurons from the mouse E16.5 cortex with AAV2/6 particles encoding each of these forms of AMPKα. Of note, we measured by real-time PCR (rt-PCR) the endogenous expression of the α1 and α2 isoforms of the catalytic AMPKα subunit. Cortical neurons were found to predominantly express the α1 subunit, as the mRNA level of AMPKα1 was found to be 9.2 ± 1.2 fold higher than that of α2 (Additional file 3: Fig. S2a). In the adult rat SN, the transcript of the α1 subunit was 85.0 ± 11.6 fold more abundant than α2 (Additional file 3: Fig. S2b). Hence, the α1 subunit is likely to be the main catalytic component of the AMPK complex both in cortical neurons and in the rat ventral midbrain.

To determine the effect of human α-syn accumulation on AMPKα activity, primary neurons were co-transduced with an AAV2/6 vector encoding human α-syn (AAV-α-syn, 3.9E7 TU). In each control condition, a similar non-coding AAV2/6 vector was added to the primary neurons to reach the same total dose of vector.

To assess the overall AMPK activity, we analyzed via western blotting the levels of total AMPKα, T172 phospho-AMPKα (pAMPK), pACC and total α-syn (Fig. 1b-d). Using a pan-AMPKα antibody, we could detect a clear increase in the total level of AMPKα for all conditions in which a variant of AMPKα was overexpressed (Fig. 1b, c). Subsequently, we analyzed the levels of T172 phosphorylation (Fig. 1b, d). An increase in T172 pAMPK was mainly observed in neurons overexpressing AMPKα1, indicating that α1 is the subunit which is the most efficiently phosphorylated in these neurons. Of note, pAMPK was nearly totally suppressed in neurons transduced with the T172Dα1 vector, suggesting that endogenous phosphorylated AMPKα was no more part of the complex when T172Dα1 was overexpressed (Fig. 1b, d).

To determine how each of these variants affects AMPK activity, we analyzed the level of pACC, a product of AMPK kinase activity. The level of pACC was decreased in neurons expressing the T172Dα1 and K45Rα2 variants, which is consistent with the lower (T172Dα1) and abolished (K45Rα2) catalytic activity of these mutants (Fig. 1b and Additional file 4: Fig. S3). Therefore, both the T172Dα1 and K45Rα2 variants act as dominant negative regulators of AMPK activity, although the T172Dα1 variant is expected to maintain low constitutive activity. There was no increase in the level of pACC level in neurons chronically overexpressing the active AMPKα subunits.

Co-infection with AAV-α-syn led to overexpression of the human α-syn protein. Total α-syn protein levels were similar in each of the conditions co-overexpressing AMPKα subunits, indicating that AMPK activity does not have any major effects on the total level of overexpressed α-syn in vitro (Fig. 1b). Next, we assessed if the overexpression of human α-syn led to any changes in the AMPKα levels in primary neurons. Remarkably, neurons accumulating human α-syn had a significant reduction in the level of total AMPKα in all conditions in which the α subunit was overexpressed (F_6,14_ = 5.754) (Fig. 1b, c). The level of endogenous AMPKα was not affected by α-syn overexpression in the control and 1-310α2 conditions. We did not observe any overall change in the level of pAMPK when α-syn was overexpressed, indicating that α-syn did not have any major effect on T172 phosphorylation despite lower levels of total AMPKα.

Overall, these results show that the accumulation of human α-syn in neuronal cells leads to a general reduction in the total level of the overexpressed AMPKα subunit, which may have implications on the activity of the complex in specific subcellular locations. However, the global level of pAMPK in basal conditions does not seem to be affected by α-syn. By over-expressing various forms of AMPKα in neuronal cells, we can modulate the formation of pAMPK and thereby the activity of the complex.

### Overexpression of the AMPKα2 subunit protects dopamine neurons from α-syn toxicity in vivo, in an AMPK complex dependent manner

**Fig. 2:**
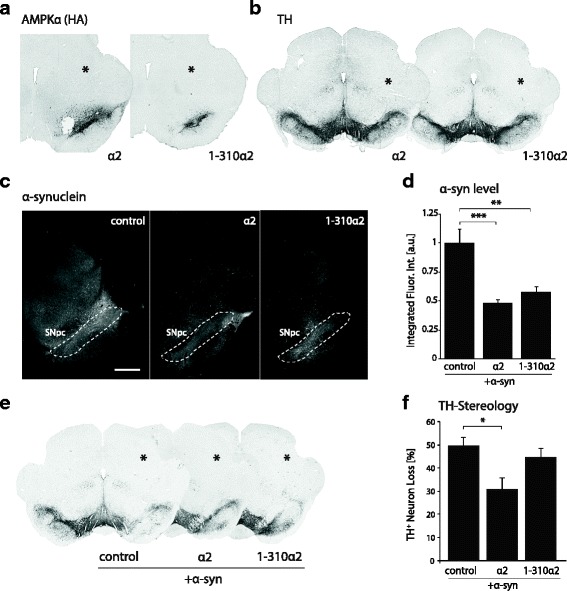
Overexpression of AMPKα2, but not the truncated 1-310α2 variant, has neuroprotective effects against α-syn in vivo*.* **a** Immunostaining for HA-tag shows the induced overexpression of either AMPKα2 or the 1-310α2 variant in the rat SN, 1 month after vector injection. **b** TH immunostaining of the SN, 1 month after AAV-AMPKα2 vector injection. Note that AMPKα2 overexpression does not cause any loss of dopaminergic neurons. **c** Immunofluorescent staining for α-syn showing overexpression in the SNpc, 4 months after co-injection of the α-syn and AMPKα vectors. Scale bar: 1 mm. **d** Relative quantification of integrated α-syn immunofluorescence intensity in the midbrain, near the site of vector injection. Note the significant decrease in α-syn abundance in the groups co-injected with a vector encoding AMPKα2 or the 1-310α2 variant. **e** Representative TH immunostaining of the SN. **f** Stereological quantification of the loss of TH-positive neurons in the SNpc. Data represent the percentage loss of neurons compared to the non-injected hemisphere. Note the significant neuroprotective effect of overexpressing the AMPKα2 subunit in the SN. *Statistical analysis*: one-way ANOVA with Tukey’s HSD post hoc test; for c: *n* = 5 (control), *n* = 6 (α2), n = 5 (1-310α2); for f: *n* = 9 (control), *n* = 11 (α2), *n* = 7 (1-310α2); *P < 0.05; **P < 0.01; ***P < 0.001. In panels a, b and e: *indicates the injected hemisphere.

We sought to establish if overexpression of wild-type form of AMPKα subunit could be neuroprotective against α-syn toxicity in vivo. First, AAV2/6 vectors encoding either the wild-type α2 subunit or the truncated 1-310α2 form, which does not integrate in the AMPK complex and carries constitutive catalytic activity, were tested for expression of AMPKα following injection in the ventral midbrain (1.2E6 TU). Expression of both AMPKα2 variants (HA-tag staining) was detectable in the SNpc at one month post-vector injection (Fig. 2a). Furthermore, tyrosine hydroxylase (TH) immunostaining showed that there was no evident loss of dopaminergic neurons in the SNpc following overexpression of AMPKα2 alone (Fig. 2b).

To assess the potential neuroprotective effects of AMPKα2 overexpression, rats were unilaterally co-injected in the SNpc with the AAV-α-syn vector (1.5E7 TU) as previously described [35], together with either a control non-coding vector (1.2E6 TU), or with each of the two vectors overexpressing the AMPKα2 subunit at the same vector dose. Immunostaining for α-syn, 4 months after vector injection, confirmed overexpression of human α-syn in the SN in all conditions (Fig. 2c). To assess α-syn abundance, we quantified its level of expression in the midbrain by immunofluorescence. Compared to the animals co-injected with the AAV-α-syn and control vectors, we found a significant reduction in the α-syn level when either AMPKα2 or the truncated 1-310α2 form were co-expressed (F_2,13_ = 15.64) (Fig. 2c, d).

The survival of the nigral neurons was analyzed four months after injection, by stereological counting of neurons positive for TH in the SNpc. Compared to the non-injected hemisphere, overexpression of human α-syn led to a 49.4 ± 3.9% loss of nigral TH-positive neurons (Fig. 2e, f). Remarkably, overexpression of the AMPKα2 subunit showed a significant neuroprotective effect on TH-positive neurons, as the α-syn-induced loss was decreased to an average value of 30.7 ± 4.9% in this group (F_2,24_ = 5.187). In contrast, the truncated 1-310α2 variant, which has constitutive activity but does not integrate into the AMPK complex, did not induce any significant effect on TH-positive neuron loss (Fig. 2e, f).

Overall, overexpressing AMPKα reduces the α-syn level in the midbrain, and AMPKα2 has neuroprotective effects on dopamine neurons accumulating human α-syn. The observed neuroprotection depends on the incorporation of the active subunit into the heterotrimeric AMPK complex, suggesting that the catalytic activity of AMPK may have to be coupled with local sensing of the energy status in order to grant protection.

### Overexpression of AMPKα2 subunit increases mitochondrial size and mitochondrial mass in vivo

**Fig. 3:**
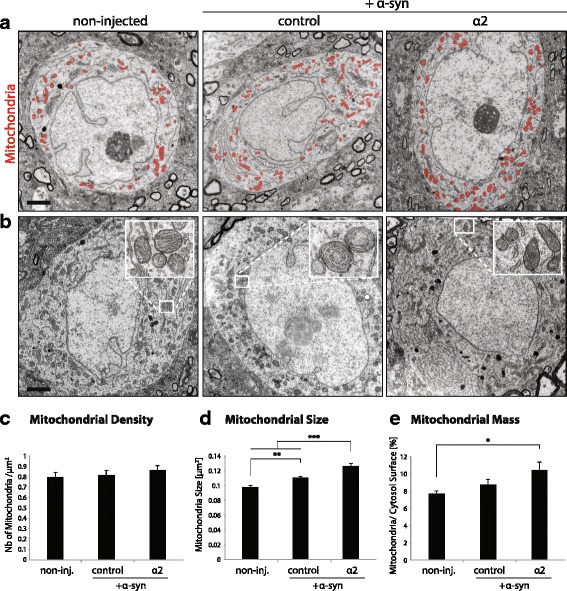
AMPKα2 overexpression improves mitochondrial morphology in nigral neurons. **a** Representative TEM pictures of neurons in the SNpc, co-injected either with non-coding (‘control’) and α-syn vectors, or with AMPKα2 and α-syn vectors. Neurons in the non-injected hemisphere are also shown for comparison. Animals are analyzed at 1 month after vector injection. Mitochondrial network is marked in red. Scale bar: 2 μm. **b** Representative pictures of nigral neurons from the same three conditions, with high-magnification insets showing mitochondrial morphology. Note the abnormal mitochondrial morphology in neurons from the SNpc injected with the AAV-α-syn vector, with concentric cristae. **c** Mitochondrial density, expressed as the number of mitochondria per cytosol μm^2^. **d** Mitochondrial size. Note the statistically significant increase in mitochondrial size in neurons co-expressing AMPKα2 and α-syn, as compared to other conditions. **e** Mitochondrial mass expressed as the percentage of cytosolic surface area occupied by mitochondria. Note the significant increase in the mitochondrial mass in neurons co-expressing AMPKα2 and α-syn. *Statistical analysis*: one-way ANOVA with Newman-Keuls post hoc test; non-injected: *n* = 23 neurons, with total number of 1504 mitochondria; α-syn + non-coding: *n* = 26 neurons, with total number of 1962 mitochondria; α-syn + AMPKα2: n = 23 neurons, with total number of 1974 mitochondria. For each condition, nigral neurons were analyzed in samples obtained from two separate animals; *P < 0.05; **P < 0.01; ***P < 0.001

To further explore the effects of AMPKα2 overexpression in vivo, we examined mitochondrial morphology in nigral dopamine neurons. Using transmission electron microscopy (TEM), we investigated the status of mitochondria after one month of AMPKα2 and α-syn co-overexpression (Fig. 3a, b), when nigral neurodegeneration is still mild. Neurons in the SNpc injected with the α-syn-encoding and non-coding vectors were compared with the non-injected contralateral side and with the SNpc in rats co-injected with the α-syn- and AMPKα2-encoding vectors. We analyzed the relative number of mitochondria per μm^2^ of cytosol (mitochondrial density) for each group. For this parameter, there was no significant difference across conditions (Fig. 3c). However, we noticed that only in the SNpc injected with the vector encoding human α-syn, some neurons (27% of the neurons analyzed) displayed abnormal mitochondrial morphology, mainly characterized by concentric circles of cristae membranes (Fig. 3b). Compared to the SNpc injected with AAV-α-syn, mitochondrial morphology was improved following co-injection of the AMPKα2-expressing vector, with a decrease in the overall proportion of neurons that displayed abnormal cristae (9% of the neurons analyzed). Furthermore, we observed a significant increase in the size of mitochondria (Fig. 3d) (F_2,5437_ = 22.5), as well as an increase of the mitochondrial area fraction per neuron (Fig. 3e) (F_2,69_ = 4.23). The effects related to mitochondrial size and area fraction were even more pronounced when compared to neurons in the non-injected SNpc.

All in all, in vivo overexpression of α-syn has clear effects on mitochondrial morphology and causes a significant increase in average mitochondrial size. Co-injection with the AMPKα2-encoding vector improves mitochondrial morphology and increases mitochondrial mass in nigral neurons.

### Overexpression of the T172Dα1 AMPK variant provides the most effective neuroprotection against the toxic effects of α-syn accumulation

The possibility to provide neuroprotective effects against α-syn by increasing the capability of nigral dopamine neurons to sense energy status via AMPKα overexpression was further tested by comparing the α1 and α2 subunits. We also included the T172D variant of the α1 subunit, which is expected to provide a low but constitutive AMPK activity. The paradigm was similar to the previous experiment, based on the unilateral injection of AAV-AMPKα vectors at the same time as the pathogenic AAV-α-syn vector in the rat SNpc.

**Fig. 4:**
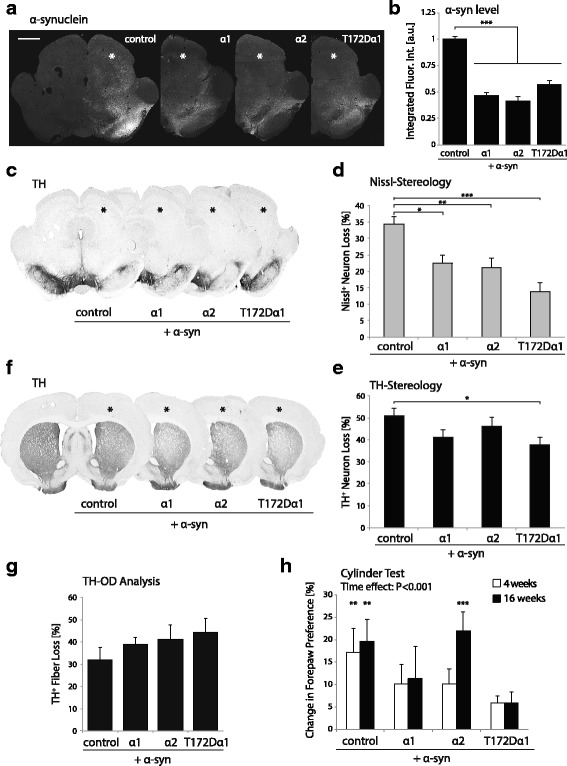
Overexpression of the AMPK T172Dα1 subunit has the most significant neuroprotective effect against α-syn toxicity in vivo*.* AMPKα1, α2 or T172Dα1 subunits provide significant neuroprotection against α-syn toxicity in vivo*,* 4 months after vector injection. **a** Immunofluorescent staining for α-syn showing overexpression in the SNpc, 4 months after co-injection of the α-syn and AMPKα vectors. Scale bar: 1 mm. **b** Relative quantification of integrated α-syn immunofluorescence intensity in the midbrain, near the site of vector injection. Note the significant decrease in α-syn abundance in the groups co-injected with a vector encoding AMPKα1, α2 or the 1-310α2 variant (n = 5 per group). **c** Representative TH immunostaining of the SN. **d** Stereological evaluation of the loss of Nissl-stained neurons in the SNpc shows a significant neuroprotective effect for all AMPKα subunits. **e** Stereological analysis of the loss of TH-positive neurons in the injected SNpc. Note the significant neuroprotection induced by overexpression of the T172Dα1 subunit. **f** Representative TH immunostaining of the STR. **g** Optical densitometry analysis of TH-immunoreactivity in the STR, expressed as the percentage loss versus the non-injected hemisphere. **h** Spontaneous behavior of animals in cylinder test, expressed as the change in the preference of forepaw use. *Statistical analysis*: one-way ANOVA with Newman-Keuls post hoc test; for stereological and OD analysis of TH-immunoreactivity, *n* = 8 (control), n = 11 (α1), n = 8 (α2), n = 8 (T172Dα1); for cylinder test, repeated measures two-way ANOVA; n = 9 (control), *n* = 14 (α1), *n* = 10 (α2), n = 11 (T172Dα1); *P < 0.05; **P < 0.01; ***P < 0.001. In panels a, c, f: * indicates the injected hemisphere

The extent of α-syn-induced toxicity was evaluated at four months after vector injection. The abundance of α-syn in the midbrain was assessed by fluorescence immunohistochemistry (Fig. 4a), and quantification of the α-syn level near the site of vector injection again revealed a significant decrease in the groups co-injected with AAV encoding active forms of AMPKα, including the T172Dα1 variant (F_3,16_ = 59.6) (Fig. 4b). The total number of neurons with dopamine morphology present in the SNpc was estimated using TH and Nissl co-staining (Fig. 4d). Remarkably, the protective effect on the number of Nissl-positive neurons was significant in all AMPKα overexpressing conditions (F_3,31_ = 9.955), and no major difference was observed between the α1 and α2 subunits. The effect was most evident for the T172Dα1 variant, with an average loss of Nissl-positive neurons of only 14.2 ± 2.9%, compared to 31.9 ± 2.5% for the control group co-injected with AAV-α-syn (Fig. 4d). Next, we quantified the loss of TH-positive neurons in the SNpc (Fig. 4e). There was a trend towards a protective effect with the α1 and α2 subunits. However, only overexpression of the T172Dα1 variant induced a statistically significant protection. The loss of TH-positive nigral neurons was decreased to 38.1 ± 3.2%, with respect to 49.1 ± 3.5% for the control non-coding vector (Fig. 4e). The reduced neuroprotective effects observed when measuring the number of TH-positive neurons, as compared to the total number of Nissl-positive neurons in the SNpc, indicates a likely down-regulation of TH expression in neurons overexpressing AMPKα. This was further confirmed by measuring TH immunoreactivity in the STR (Fig. 4f, g). Indeed, no protection was observed at the level of TH-positive fiber loss between the groups (Fig. 4g). Down-regulation of the dopamine markers was further confirmed using immunohistochemistry for the dopamine transporter (DAT) in the STR (Additional file 5: Fig. S4a). Optical density of the DAT signal was decreased to a similar extent as compared to TH (Additional file 5: Fig. S4b).

The development of asymmetric spontaneous motor behavior was assessed using the cylinder test (Fig. 4h). Animals from the control group injected with the AAV-α-syn vector showed a progressive increase in preferential right forepaw use, reaching 17.2 ± 5.3% at week 4 and 19.6 ± 4.9% at week 16. The gradual motor impairment due to α-syn overexpression was statistically significant (time effect: F_2,40_ = 22.440). In contrast, the rats in the T172Dα1 group did not show any significant progression of motor asymmetry over time, reaching only 6.0 ± 2.4% of preference for the non-affected side at week 16 (Fig. 4h). Although the difference between these two groups did not reach significance, the lack of progression of motor asymmetry in the T172Dα1 animals indicates a preserved motor function following unilateral injection of the α-syn-encoding vector.

Summarizing, overexpression of AMPKα subunits provides a significant neuroprotection of dopamine neurons in SNpc against α-syn-driven toxicity. This effect is not dependent on the isoform of the catalytic subunit being overexpressed, since both AMPKα1 and α2 showed a similar level of protection. Nevertheless, the most prominent neuroprotective effect was obtained via overexpression of AMPK T172Dα1 subunit. Surprisingly however, it did not coincide with a protection of TH-positive neurons or striatal fibers, which hints to a possible down-regulation of dopamine marker expression in the surviving neurons. Nevertheless, overexpression of the T172Dα1 subunit showed an effective rescue of motor behavior, which suggests a functional preservation of the dopamine nigrostriatal function.

### T172Dα1 AMPK variant decreases the presence of dystrophic axons in the STR

**Fig. 5:**
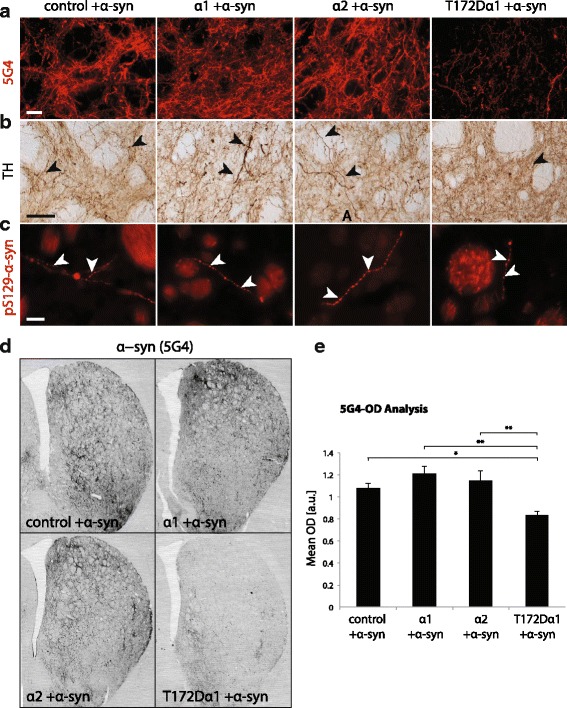
T172Dα1 overexpression decreases the α-syn burden in the striatum. Deposition of human α-syn in dystrophic axons is analyzed at 4 months after vector injection. **a** Staining for aggregated forms of α-syn (5G4 antibody) in the STR. Note the significantly lower signal in the representative animal that overexpresses T172Dα1 AMPK. Scale bar: 50 μm. **b** TH-positive dystrophic fibers (arrowheads) in the STR. Note the correlation with the signal for aggregated forms of α-syn. Scale bar: 50 μm. **c** Dopamine fibers in the STR, positive for phosphorylated form of α-syn (pS129-α-syn). Arrowheads indicate characteristic fibers’ dystrophy. Scale bar: 25 μm. **d** Representative sections of the medial STR stained for aggregated forms of α-syn (5G4 antibody). Note the lower signal in the animal overexpressing the T172Dα1 subunit. **e** Optical densitometry for the 5G4 signal, showing a significant reduction of α-syn burden in the STR of rats co-expressing human α-syn and AMPK T172Dα1 in the nigrostrialal system. *Statistical analysis*: one-way ANOVA with Newman-Keuls post hoc test; for 5G4-OD analysis, n = 8 (control), *n* = 12 (α1), n = 8 (α2), n = 8 (T172Dα1); *P < 0.05; **P < 0.01; ***P < 0.001

Next, we explored how different forms of AMPKα affect the formation of dystrophic dopaminergic fibers projecting to the STR, characterized by the pathologic accumulation of α-syn. We performed an immunofluorescent staining using the 5G4 anti-α-syn monoclonal antibody, which specifically recognizes pathological species of aggregated α-syn [36]. Within the STR, the signal for 5G4 was observed in axonal fibers, which often appeared dystrophic (Fig. 5a). These dystrophic fibers were also evident in TH immunostaining (Fig. 5b).

Phosphorylation of α-syn is considered as a marker for the pathological deposition of this protein, as the majority of Lewy bodies contain α-syn phosphorylated on residue S129 (pS129-α-syn) [37]. Immunostaining of STR sections revealed the presence of dystrophic fibers positive for pS129-α-syn, with a morphology closely resembling that of 5G4-positive dystrophic fibers. Despite being detected in all conditions, they appeared however to be less abundant than their 5G4 counterparts (Fig. 5c).

The abundance of dystrophic fibers was particularly high in the AMPKα1 and α2 injected rats, and appeared to be reduced in the injected hemisphere of the T172Dα1 rats (Fig. 5d). To quantify the effects of AMPK on dystrophic axonal fibers, we performed an optical densitometry (OD) analysis of the 5G4 signal in the dorsal part of the STR. Remarkably, the mean 5G4 fluorescence intensity was significantly decreased in rats overexpressing the T172Dα1 variant, compared to all three other groups (F_3,32_ = 6.4) (Fig. 5e). By contrast, in the AMPKα1 condition, the mean intensity appeared slightly higher than in the control group, although the difference was not significant. Overall, these results indicate that AMPK T172Dα1 overexpression, which leads to constitutive low AMPK activity, significantly reduces the density of dystrophic dopamine fibers containing aggregated α-syn, as compared to all other conditions.

Overall, AMPK T172Dα1 overexpression leads to a significant reduction in the abundance of dystrophic fibers that contain aggregated (5G4-positive) α-syn, as compared to all other conditions. These results indicate that the constitutive low AMPK activity conferred by overexpressing the T172Dα1 variant has protective effect on the development of α-syn pathological features in the STR. However, other mechanisms are likely to account for the protective effects of AMPKα1 and α2.

### In cortical neurons, AMPKα overexpression limits the accumulation of autolysosomes caused by human α-syn

To further explore the effects of AMPKα on autophagic and mitochondrial activity, we next conducted in vitro experiments using mouse primary cortical neurons induced to overexpress human α-syn. As it is well established that AMPK controls autophagic activity [38, 39], we explored the effect of the different forms of AMPKα on autophagic and lysosomal markers at steady state, either in the absence or presence of human α-syn.

**Fig. 6:**
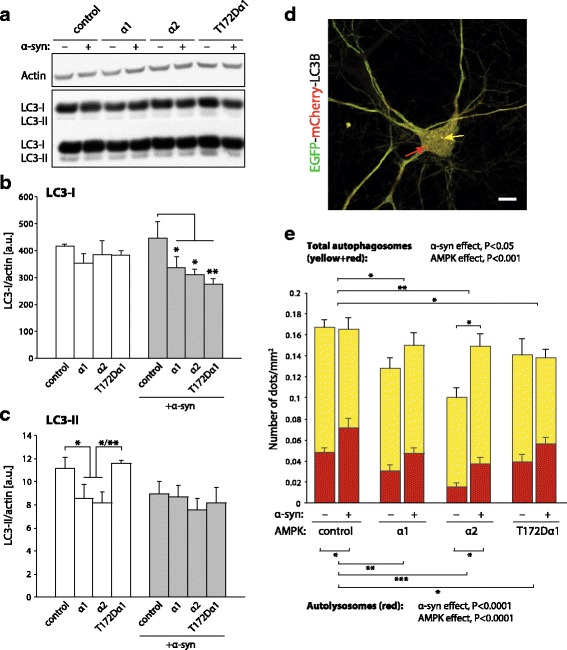
AMPKα reduces lysosome accumulation in primary cortical neurons overexpressing α-syn. **a** Primary cortical neurons transduced with α-syn-encoding vector (or non-coding vector as control) are co-transduced with AMPKα expressing vectors. Protein analysis by western blotting shows LC3-I, LC3-II and actin. Signal intensity is enhanced in the lower panel to show LC3-II immunoreactivity. **b** Relative quantification of LC3-I levels normalized to actin. AMPKα overexpression decreases LC3-I level in neurons expressing human α-syn. **c** Relative quantification of LC3-II levels normalized to actin. Note a significant decrease in LC3-II levels in neurons overexpressing AMPKα in the absence of human α-syn. **d** Representative image of a cortical neuron expressing the fusion protein LC3B-EGFP-mCherry. Note the presence of yellow (autophagosomes, yellow arrow) and red vesicles (autolysosomes, red arrow) in the cytosol. Scale bar: 10 μm. **e** Quantification of the average number of autophagosomes and autolysosomes per cell, normalized to cytosol area. Note the reduction in the total number of autophagic vesicles, in neurons overexpressing either AMPKα1 or α2. Overexpression of α-syn increases the number of autolysosomes in neuronal cells. AMPKα reduces the number of autolysosomes, even in presence of α-syn. *Statistical analyses*: (b, c) repeated measures two-way ANOVA with Fisher‘s LSD post hoc test; *n* = 3 per condition. (e) two-way factorial ANOVA with Newman-Keuls post hoc test; b, c: for each condition *n* = 22–30 neurons from 3 separately infected wells; *P < 0.05; **P < 0.01; ***P < 0.001

As a first indicator, we assessed by western blotting the level of the microtubule-associated protein 1B light chain 3 (LC3-I), and LC3-II, its lipidated form associated to autophagosome formation (Fig. 6a). Overexpression of human α-syn did not have any significant effect on the levels of LC3-I and LC3-II in the control condition (Fig. 6b, c). However, we noticed that α-syn accumulation modified the expression of these autophagic markers in neurons overexpressing AMPKα. In neurons that do not overexpress α-syn, LC3-I expression remained very similar across conditions, regardless of the overexpression of the AMPKα subunits (Fig. 6b). In contrast, the level of LC3-II was significantly reduced in neurons overexpressing either AMPKα1 or α2 (F_3,12_ = 3.98) (Fig. 6c). In contrast, when human α-syn was present, we observed a significant reduction in the LC3-I level in neurons overexpressing AMPKα, which was most pronounced with the constitutively active T172Dα1 variant (F_3,12_ = 4.0) (Fig. 6c). The level of LC3-II however remained similar across conditions. These results indicated an adaptive response to α-syn in neurons overexpressing AMPKα.

Accumulation of α-syn can affect autophagic activity and is associated with perturbations of the lysosomal function [40–43]. To determine the effects of α-syn on the distribution of LC3-II between autophagosome and lysosome vesicles, primary cortical neurons were co-transduced with a reporter construct encoding LC3B fused with mCherry and EGFP (LC3B-mCherry-EGFP described in [29]). Yellow puncta positive for both mCherry and EGFP indicate autophagosomes, which have not yet fused with lysosomes, whereas red puncta are characteristic for autolysosomes (Fig. 6d). Consistent with the effect observed for LC3-II (Fig. 6c), the total number of puncta was found to be lower in the conditions in which the AMPKα1 and α2 subunits were overexpressed, as compared to the control condition. This effect was statistically significant for the α2 subunit (α-syn x AMPK effect: F_3,208_ = 2.4) (Fig. 6e).

Remarkably, human α-syn overexpression was found to increase the number of red puncta, which indicates an abnormal accumulation of autolysosomes (α-syn effect: F_1,208_ = 22.08) (Fig. 6e). Although the effect of α-syn on the number of autolysosomes was observed in all conditions, the number of red puncta remained lower in neurons overexpressing AMPKα1 or α2 (Fig. 6e). In neurons overexpressing AMPKα2, the number of autolysosomes was still reduced by 48% when compared to the control α-syn condition.

Importantly, there was no evidence that the effect of AMPKα was associated to any downregulation of autophagic activity in neurons overexpressing α-syn. Indeed, both the total number of autophagic vesicles (Fig. 6e), as well as the number of autophagosomes positive for both mCherry and EGFP (Additional file 6: Fig. S5), remained similar in neurons co-expressing α-syn and AMPKα as compared to the control α-syn condition.

These results indicate that human α-syn causes an accumulation of autolysosomal markers, which may reflect defective lysosomal enzymatic activity, as previously reported [41, 44]. However, in neurons overexpressing either AMPKα2 or AMPKα1, albeit to a lesser extent, the number of autophagic vesicles was increased when human α-syn was overexpressed (Fig. 6e). This effect coincides with a significant reduction in the number of autolysosomes, as compared to neurons overexpressing α-syn only. In contrast, α-syn did not induce any change in the total number of autophagic vesicles in neurons overexpressing the constitutively active T172Dα1 variant (Fig. 6e). Overall, AMPKα contributes to controlling the accumulation of autolysosomes in neurons overexpressing α-syn.

### AMPKα increases mitochondrial mass in neurons overexpressing human α-syn

AMPKα2 overexpression has significant effects on the mitochondrial network in vivo (see Fig. 3). To further characterize the combined effects of AMPKα and α-syn on mitochondria, we determined the mitochondrial mass and mitochondrial DNA (mtDNA) quantity in cortical neurons overexpressing each of the AMPKα variants in vitro. In parallel, the neurons were challenged with α-syn overexpression, which has been reported to affect the turnover as well as the dynamics of mitochondria [45, 46].

**Fig. 7:**
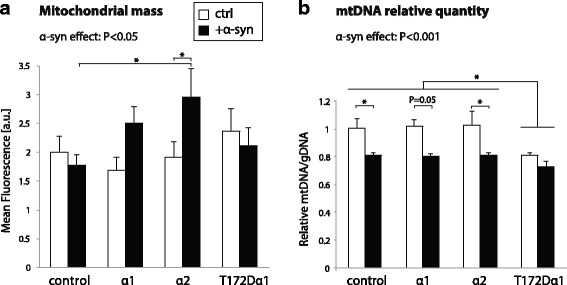
Effects of AMPKα and α-syn on mitochondria in cortical neurons. **a** Average mitoDsRed fluorescence intensity measured by flow cytometry in individual neuronal cultures. Note the significant increase in the mitoDsRed fluorescence intensity in neurons co-expressing AMPKα2 and α-syn, as compared to the control. **b** Real-time PCR quantification of mtDNA (16S) versus cellular gDNA (Hk2). Note that α-syn overexpression results in overall decline in the relative amount of mtDNA per cell. In cortical neurons overexpressing AMPK T172Dα1, the amount of mtDNA is significantly lower, regardless of α-syn being overexpressed. *Statistical analysis*: two-way factorial ANOVA with Newman-Keuls post hoc test; for mitochondrial mass, n = 10–12 per condition; for mtDNA, *n* = 4 per condition, except for α1 + α-syn (n = 3); *P < 0.05

First, we determined the mitochondrial mass by overexpressing the mitoDsRed probe in neuronal cultures and measuring fluorescence intensity using flow cytometry (Fig. 7a). In control neurons, we found that α-syn overexpression did not induce any change in the total mitochondrial load. However, neurons overexpressing AMPKα1 and α2 responded to α-syn by increasing the average mitoDsRed fluorescence per cell (F_1,86_ = 4.87) (Fig. 7a). The increase in mitoDsRed fluorescence reached statistical significance in neurons overexpressing the AMPKα2 variant (Fig. 7a), which is in line with the aforementioned increase in mitochondrial mass observed in vivo (see Fig. 3f). Remarkably, this effect was not observed in neurons expressing the constitutively active T172Dα1 variant (Fig. 7a), which further supports the role of fully active and responsive AMPKα subunits in sensing α-syn-induced changes.

Next, we measured the relative quantity of mtDNA per cell by rt-PCR, seven days after vector transduction (Fig. 7b). Overexpression of α-syn induced a significant decrease in the cellular quantity of mtDNA (F_1,23_ = 25.367). Remarkably however, the α-syn-induced difference was attenuated in neurons overexpressing AMPK T172Dα1, mainly because the amount of mtDNA was also decreased in the absence of α-syn overexpression (F_3,23_ = 3.8) (Fig. 7b).

To determine the overall effects on metabolic activity, we sought to investigate mitochondrial respiration in primary cortical neurons co-overexpressing AMPKα and α-syn. We measured both respiration in basal conditions and following exposure to CCCP, a mitochondrial uncoupling agent used to assess maximal respiration (Additional file 7: Fig. S6). Overexpression of the AMPKα variants per se did not have any major effects on basal neuronal respiration, except for a slight reduction in oxygen consumption rate (OCR) in neurons overexpressing AMPKα1. Even though overexpression of human α-syn had only mild effects on OCR, we did notice an overall statistically significant increase of basal OCR and reduced reserve respiratory capacity. The effect on the reserve capacity was particularly evident in neurons overexpressing AMPKα. However, in neurons expressing the constitutively active T172Dα1 variant, the reserve capacity was similarly decreased both in the presence and absence of α-syn. AMPKα does not induce any major increase in mitochondrial activity and reduces the reserve respiratory capacity in neurons expressing human α-syn, an effect consistent with the measured mtDNA content. Overall, these results show that in primary neurons overexpressing human α-syn, AMPKα has an effect mainly on the mitochondrial mass, without increasing the mtDNA content and the respiratory capacity.

## Discussion

Overexpression of AMPKα1 or α2 subunits, which integrate into the AMPK complex, can protect dopamine neurons against the toxic effects of human α-syn accumulation in vivo. In particular, the T172Dα1 variant, which is characterized by low chronic catalytic activity, provides the most effective neuroprotection of dopamine neurons following unilateral injection of the α-syn-expressing AAV vector. Furthermore, T172Dα1 significantly decreases the number of dystrophic axons containing aggregated forms of α-syn in the STR.

We used the same genetic approach to control AMPK activity in primary neurons and assess effects on mitochondria and autophagy markers in vitro. Overexpressing AMPKα subunits increases the mitochondrial mass and limits the accumulation of lysosomal material in neurons co-expressing human α-syn. By contrast, α-syn has only limited effects on parameters related to lysosomal and mitochondrial activity in neurons expressing the constitutively active T172Dα1 variant.

### Alpha-synuclein and AMPK activity in neuronal cells

As α-syn can perturb mitochondria [45, 47], it may lead to metabolic stress in neurons and affect AMPK signaling. To address this question, α-syn was co-expressed with different forms of human AMPKα. It has been previously reported that α-syn reduces AMPK phosphorylation in neuronal cells [26]. Although we did not observe any significant change in pAMPK level, we noticed a significant reduction of total AMPKα in mouse cortical neurons overexpressing α-syn. This effect was evident on overexpressed AMPKα, and not on endogenous AMPKα, which may indicate that the overabundance of the subunit facilitates α-syn-induced reduction of its expression level. It is therefore possible that α-syn mainly affects the fraction of the protein which is not associated to the AMPK complex, and may therefore not cause any change in pAMPK level. Although α-syn accumulation may affect AMPK levels, the absence of any increase in AMPK or ACC phosphorylation in neurons overexpressing α-syn, confirms that α-syn is a rather mild metabolic stressor, at least in vitro. Nevertheless, the effects of α-syn on neuronal metabolism may be more evident at later stages of the pathology [47].

Although we have not observed any effect of AMPKα on the level of α-syn in primary neuronal cultures, the level of α-syn is significantly reduced in the ventral midbrain when active forms of AMPKα are co-expressed with α-syn. Although one cannot rule out that AMPKα affects α-syn translation [48], it appears more likely that the down-regulation of α-syn expression is caused by increased degradation of the protein. AMPK has been shown to enhance protein degradation in various tissues, via mechanisms that involve either the ubiquitin proteasome pathway or autophagy [49–51]. Although both systems are implicated in α-syn turnover, autophagic activity is mainly recruited when the α-syn burden is increased [52]. The effects of AMPK on α-syn turnover will need to be further explored.

### Overexpression of AMPKα is neuroprotective against α-syn

In normal circumstances, AMPK activity is adjusted as a function of time and location at which metabolic stress occurs [53]. In order to identify the optimal pattern of AMPK activity to counteract α-syn toxicity, we chose to overexpress four variants of the α subunit in a model of PD based on the chronic overexpression of human α-syn [35]. We show that overexpression of either the AMPKα1 or α2 subunit is able to equally protect nigral dopamine neurons against α-syn toxicity. This indicates that, at least regarding neuroprotection, there is no major difference between these isoforms, when overexpressed. Moreover, the neuroprotective effect of AMPKα is dependent on integration into the AMPK complex, as overexpression of the truncated 1-310α2 form of AMPK does not provide any protection, despite its constitutive catalytic activity.

Here, we show that in vivo overexpression of α-syn has dramatic effects on mitochondrial morphology (Fig. 3). It has been previously shown that neurons deprived of PGC-1α, exhibit significant alterations in mitochondrial cristae morphology and reduced respiratory chain complex activity following in vitro or in vivo overexpression of α-syn [54, 55]. Alpha-syn can bind to the promoter sequence of PGC-1α and cause promoter methylation, a phenomenon associated with sporadic PD cases and which can lead to decreased PGC-1α expression [55, 56]. Remarkably, co-expression of α-syn with the AMPKα2 subunit increases both mitochondrial size and mass, with a partial rescue of the morphological alterations observed in mitochondria. Whether these effects are due to activation of PGC-1α and increased mitochondrial biogenesis remains to be further explored.

The neuroprotective effect of AMPKα is particularly evident when assessing the number of nigral neuronal cell bodies using Nissl staining. However, the AMPKα-induced protection of TH-positive neuronal cell bodies is less consistent, reaching statistical significance with AMPKα2 only in the first experiment (Fig. 2f), whereas being less effective in the second study (Fig. 4e). In addition, AMPKα expression has failed to protect TH- and DAT-positive axonal fibers in the STR. These results indicate that AMPKα-induced neuroprotection is most likely concomitant with an eventual decrease in the expression of dopaminergic markers. This effect is reminiscent of the phenotype of dopamine marker loss observed after chronic overexpression of PGC-1α with a postulated mechanism involving Pitx3 downregulation [30, 57].

### Most effective neuroprotection against α-syn is achieved with constitutive low AMPK activity

Remarkably, the T172Dα1 variant provides the most substantial neuroprotective effect against α-syn toxicity, mitigating the loss of Nissl-positive neurons in the SNpc by more than 50%. This form of AMPKα, which integrates into the AMPK complex, has a constitutive catalytic activity, which is independent from T172 phosphorylation. However, T172Dα1 has also a dominant negative effect on AMPK, as the activity of this variant is clearly lower than wild-type forms of AMPKα (Fig. 1b) [32, 33]. The chronic mode of low activity induced by T172Dα1 overexpression, which is likely to prevent fluctuations in AMPK activity, appears to be particularly neuroprotective against α-syn-induced toxicity.

Regarding the possible mechanism of neuroprotection, T172Dα1 variant prevents development of the α-syn pathology. Using 5G4 antibody to detect α-syn aggregates [36], we show a significant reduction of α-syn inclusions in the medial STR, only in the animals overexpressing T172Dα1 AMPK (Fig. 5e). In contrast, AMPKα1 and α2 tend to increase the deposition of aggregated α-syn and formation of dystrophic TH-positive axonal fibers. These effects are in line with previous observations that enhanced AMPK activity increases α-syn oligomers’ formation in vitro [27], although the activation of AMPK via AICAR or resveratrol was also been reported to mitigate α-syn oligomerization in a neuroglioma cell line [58]. While it remains to be determined if chronic low AMPK activity reduces the formation of α-syn oligomers in vivo, the lower level of α-syn deposition may account for the observed neuroprotective effects of the T172Dα1 variant. By preventing α-syn accumulation and aggregation, T172Dα1 overexpression could prevent the formation of dystrophic fibers in the STR and facilitate vesicular dopamine release. This might explain the improved symmetry of these animals in the cylinder test, despite the observed decrease in striatal TH immunoreactivity. However, other mechanisms are likely to account for the neuroprotective effects observed following overexpression of AMPKα1 and α2, as there is no apparent decrease in α-syn deposition in these conditions.

### The effects of AMPKα on mitochondrial and lysosomal activities

Using primary neuronal cultures, we have analyzed changes in mitochondrial and lysosomal parameters following α-syn overexpression. Cortical neurons show a significant increase in the number of autolysosomes loaded with the LC3B-mCherry-EGFP reporter when overexpressing human α-syn. This effect might reflect the accumulation of lysosomal material, possibly caused by defects in the lysosomal enzymatic activity associated with impaired trafficking of proteins [41, 44], or by perturbed lysosome recycling, as observed in neurons with defective glucocerebrosidase activity [40, 59]. At the level of mitochondria, α-syn mainly causes a decrease in the amount of mtDNA per cell, without any major effect on mitochondrial mass or respiration.

Neurons overexpressing AMPKα1 and α2 appear to be more responsive to changes induced by the overexpression of human α-syn. The observed response may not represent a rescue effect of the alterations caused by α-syn. Rather, AMPKα-overexpressing neurons may promptly undergo adaptive changes to cope with the α-syn-induced stress. AMPKα-expressing neurons respond to α-syn overexpression by increasing the total number of autophagosomes, while limiting the accumulation of autolysosomes (see Fig. 6e), which may indicate an improved autophagic process. These effects are consistent with the role of AMPK in regulating lysosome activity [60, 61]. However, further studies are warranted to determine the exact effects of AMPKα on autophagic flux in neurons accumulating α-syn.

In vivo overexpression of α-syn has dramatic effects on mitochondrial morphology in nigral neurons, as seen by electron microscopy (Fig. 3). This effect of α-syn overexpression can be rescued by AMPKα2. Furthermore, neurons exposed to α-syn-induced stress show increased mitochondrial mass when overexpressing AMPKα2, both in vitro and in vivo. These changes are likely to represent adaptations to the effects of human α-syn on mitochondria. Indeed, AMPK has been shown to promote mitochondrial biogenesis, and in the same time may promote mitochondrial fission and the autophagic turnover of defective organelles [62–64]. The overall increase in mitochondrial mass may therefore contribute to preserving mitochondrial function in neurons overexpressing α-syn. This effect could contribute to neuron survival, in particular in the dopaminergic neurons of the SNpc, which have been reported to have a low mitochondrial content [65]. It is however surprising that AMPKα is unable to rescue the decreased number of mtDNA copies observed in neurons exposed to α-syn, which may contribute to the low reserve respiratory capacity observed in these conditions. This suggests that α-syn may affect the amount of mtDNA via mechanisms that are downstream of AMPK. Nuclear α-syn has been reported to block the transcriptional activity of PGC-1α [55], which drives expression of mitochondrial transcription factor A (TFAM), a key factor in mtDNA replication [58]. Methylation of the PGC-1α promoter is also associated with sporadic PD cases [56]. Furthermore, other pathogenic factors can affect mtDNA, such as mitochondrial unfolded protein response (UPR_MT_) which leads to TFAM clearance [66], or α-syn-induced changes in the dynamics of mitochondria, such as enhanced fragmentation [45, 47]. Remarkably, an increase in mitochondrial mass has been observed in mice with a conditional knockout of *Tfam* [67, 68], suggesting that similar compensatory mechanisms may take place in neurons co-expressing α-syn and AMPKα. In addition, it is worth noting that neurons expressing the T172Dα1 variant kept constant mitochondrial mass and low mtDNA content, both in the presence and absence of α-syn. Therefore, α-syn overexpression does not seem to affect these mitochondrial parameters in neurons with constitutive low AMPK activity.

### Modulating AMPK activity: A neuroprotective approach in PD?

A growing body of evidence suggests that AMPK signaling is crucial in the process of neurodegeneration associated with many diseases [16, 69]. However, data on the potentially neuroprotective effects of AMPK signaling appear ambiguous, sometimes even contradictory [70–74]. In the context of PD, inhibition of AMPK with compound C has been shown to accelerate the process of neurodegeneration following MPTP intoxication, both in vivo [75] and in vitro [76]. On the other hand, activation of AMPK promotes neuronal degeneration in toxin models of PD, both in vivo [77] and in vitro [78], as well as in PIKE-null mice overexpressing α-syn [28]. Additionally, AMPK activation was shown to accelerate the formation of α-syn oligomers in primary neurons [27]. Overall, the beneficial effects of AMPK signaling are mainly obtained when the insult is chronic and mild, as well as slowly progressing. Conversely, in acute and severe models, such as stroke or exposure to neuronal toxins, over-activation of AMPK signaling might even exacerbate the process of neurodegeneration [53, 70, 74, 77, 78].

## Conclusions

It has been suggested that AMPK signaling progressively decreases with age [17]. Therefore, chronic activation of AMPK might serve as an effective preventive therapy when applied at the pre-symptomatic stage of neurodegenerative diseases associated with aging. Here, we provide in vivo evidence that overexpression of the AMPKα subunit can protect neurons at early stages of the α-syn pathology. In particular, the T172Dα1 variant with chronic low AMPK activity has the highest neuroprotective effects in a genetic rat model of progressive α-syn toxicity.

## Additional files

Additional file 1: Supplementary Materials and Methods. (PDF 37 kb)

Additional file 2: **Figure S1.** Levels of phospho-ACC in HEK293T cells overexpressing AMPKα. (PDF 352 kb)

Additional file 3: **Figure S2.** Levels of AMPKα1 and α2 transcripts in primary mouse cortical neurons and in the rat SN. (PDF 855 kb)

Additional file 4: **Figure S3.** Levels of phospho ACC in primary cortical neurons overexpressing AMPKα and α-syn. (PDF 222 kb)

Additional file 5: **Figure S4.** Down-regulation of dopaminergic markers in the nigrostriatal system following overexpression of human α-syn and T172Dα1 AMPK subunit. (PDF 1785 kb)

Additional file 6: **Figure S5.** Number of mCherry+/GFP+ autophagosomes in primary cortical neurons overexpressing AMPKα and/or α-syn. (PDF 221 kb)

Additional file 7: **Figure S6.** Mitochondrial respiration in cortical neurons co-expressing AMPKα and α-syn. (PDF 144 kb)

## Abbreviations

AAV: Adeno-associated virus
AID: Auto-inhibitory domain
AMPK: AMP-activated protein kinase
ANOVA: Analysis of variance
gDNA: Genomic DNA
HA: Hemagglutinin
mtDNA: Mitochondrial DNA
OCR: Oxygen consumption rate
OD: Optical densitometry
pACC: Phospho-acetyl-CoA carboxylase
pAMPK: Phospho-AMPK
PD: Parkinson’s disease
pS129-α-syn: α-syn phosphorylated on residue S129
SNpc: Substantia nigra pars compacta
STR: Striatum
tAMPK: Total AMPK
TEM: Transmission electron microscopy
TH: Tyrosine hydroxylase
TU: Transducing unit
α-CTD: Alpha subunit C-terminal domain
α-syn: Alpha-synuclein
