# Supplementary material for: Modulating the catalytic activity of AMPK has neuroprotective effects against α-synuclein toxicity
Source: Mol Neurodegener. 2017 Nov 3;12:80. doi: 10.1186/s13024-017-0220-x (PMC5670705; doi:10.1186/s13024-017-0220-x)
Supplement: Supplementary file 1 — Supplementary Materials and Methods. (PDF 37 kb) [file 13024_2017_220_MOESM1_ESM.pdf]

Modulating the catalytic activity of AMPK has neuroprotective effects against α-synuclein toxicity

Wojciech Bobela[1], Sameer Nazeeruddin[1], Graham Knott[2], Patrick Aebischer[1], Bernard L. Schneider[1,*]

Supplementary Materials and Methods

Primers

Primers used to estimate the amount of mtDNA

Name of primer	Sequence
**HK2 fw**	GCC AGC CTC TCC TGA TTT TAG TGT
**Hk2 rev**	GGG AAC ACA AAA GAC CTC TTC TGG
**16S fw**	CCG CAA GGG AAA GAT GAA AGA C
**16S rev**	TCG TTT GGT TTC GGG GTT TC

Measurement of neuronal respiration

All experiments were performed using Seahorse XF96 Extracellular Flux Analyzer. Murine primary cortical neurons were plated at a density of 40,000 cells per well and cultured in the same conditions as aforementioned. Half of the regular culture medium was exchanged 4 days and 1 day before the assay, for the Neurobasal-A medium (Thermofisher Scientific #10888-022) supplemented with 2 mM glucose, 20 mM pyruvate (Sigma #P5280), 2 % B27, 1 % GlutaMax and 1 % Penicillin/Streptomycin. On the day of oxygen consumption rate (OCR) measurements, volume of medium in each well was normalized to 150 µl. Measurements of basal oxygen consumption rate were repeated four times, every 8 min, after which 20 µM CCCP (Abcam #ab141229) was applied in order to establish maximal respiration and determine reserve respiratory capacity, which was defined as the ratio between maximal respiration and the average of the four measurements of basal OCR. When required, on the fifth day of culture neurons were infected with a corresponding AAV vector at a dose of 3E5 TU. Evaluation of respiration was performed at day seven post-infection.

Biochemical analysis of protein expression

All experiments were performed using 4-12 % gradient polyacrylamide gels (Thermofisher Scientific #NP0335). 25 µg of protein sample were resuspended in 25 µl of loading buffer and loaded onto the gel. Following electrophoresis run (200 V for 35 min), proteins were transferred onto a nitrocellulose membrane at 100 V for 60 min, using wet transfer technology. A PVDF membrane was used for LC3 immunoblotting. Membranes were briefly washed in PBS containing 0.1 % Tween (PBS-Tween) and stained using Red Ponceau solution to estimate transfer efficiency. Membranes were incubated for 1 hr at room temperature in the 5 % non-fat dry milk (Bio-Rad #1706404) solution and at 4°C overnight with primary antibody. On the following day, membranes were washed 3 times for 15 min each, in the PBS-Tween solution, and incubated for 1 hr at room temperature with the secondary antibody conjugated to horseradish peroxidase. After three washing steps, the signal was detected with Western ECL Blotting Substrate (Bio-Rad #1795060) and the light emission was analyzed using a ChemiDoc XRS system (Bio-Rad). For detection of other proteins of interest, the same membrane was incubated for 22 min at 37°C in the Restore Western Blot Stripping Buffer (Thermofisher Scientific #21059).

Stereotaxic injection of viral vectors, preparation of brain tissues and immunohistochemistry

For stereotaxic injections, animals were anaesthetized using a mix of Narcoxyl 2 (10 mg/kg intraperitoneally) and Ketaminol 10 (75 mg/kg intraperitoneally) and subsequently placed in the stereotaxic frame (David Kopf instruments). Using Hamilton syringe with a 34-gauge blunt tip needle operated by an automatic injection pump (CMA Microdialysis), 2 µl of vector suspension was injected at the speed of 0.2 µl/min. The needle was left in place for additional time of 5 min before being slowly withdrawn from the injection site.

Animals were sacrificed, their brains perfused with 4 % PFA and post-fixed for 1.5 h in 4 % PFA. After post-fixation, samples were transferred to the 25 % sucrose solution and kept at 4°C. Brains were cut at a thickness of 25 µm using a microtome. All sections were kept at 4°C in PBS supplemented with azide to prevent potential contamination.

Immunofluorescent staining: nigral and/or striatal sections were washed in PBS and incubated in blocking solution (10 % serum and 0.1 % Triton X-100) for 2 h at room temperature. After blocking, sections were incubated overnight at 4°C with the primary antibody in 5 % serum, 0.1 % Triton X-100. After three washing steps for 10 min in PBS, sections were incubated with the secondary antibody for 2 h at 4°C, and washed again in PBS. Subsequently, sections were mounted, sealed with Mowiol and kept at 4°C protected

from light for further analysis. For the DAT immunostaining, we performed an antigen-retrieval procedure before immunostaining, by incubating mounted sections in 10 mM trisodium citrate buffer (pH 6) for 20 min at 95°C.

DAB immunostaining: nigral and/or striatal sections were washed in PBS and incubated for 1 hr at 37°C in 0.1 % phenylhydrazine to quench endogenous peroxidase activity. After three washing steps in PBS, sections were incubated in PBS with 10 % serum and 0.1 % Triton X-100 for 2 h at room temperature. After blocking, sections were incubated overnight at 4°C (for 48 hrs at 4°C for the striatal sections) with the primary antibody resuspended in 5 % serum and 0.1 % Triton X-100. After three washing steps, sections were incubated with the secondary antibody for 2 h at 4°C, and washed again in PBS. Incubation with ABC solution (Vector Laboratories #PK-6100) and DAB revelation (Thermofisher Scientific #34065) were performed according to manufacturer's instructions. Stained sections were mounted, dried and sealed with Eukitt medium (O. Kindler) and kept at room temperature for further analysis.

Stereological evaluation of neuron loss

Loss of dopamine neurons was evaluated in 25 µm rat midbrain sections using the Stereo Investigator v.8 software (MBF Biosciences), adapting the following measurement criteria: grid size (180x200 µm); counting frame (75x75 µm); guard zones (2 µm). Dopamine neurons were counted in every 6th section covering the stretch of entire SNpc, using the 60x oil objective. Two rounds of measurements were carried out. In the first round, we performed a counting of the TH-positive cell bodies. In the second round, neurons in the SNpc with morphological features specific to dopamine neurons, i.e. pale cytosol, large nucleus and at least two nucleoli, were visualized by Nissl staining, identified and counted. The results of dopamine neurons estimation are presented as % loss of neurons in the injected SNpc, compared to the non-injected side.

Transmission electron microscopy

Anesthetized rats were perfused with a solution of 2.5 % glutaraldehyde and 2 % paraformaldehyde in 0.1 M phosphate buffer, pH 7.4 for 2 h. Following perfusion, the brains were removed and cut coronally (80 µm thickness) at the level of SNpc, using a vibratome. Subsequently, sections were washed in 0.1 M cacodylate buffer and post-fixed for 40 min each, first in the 1.5 % potassium ferrocyanide, then in 1 % osmium tetroxide, and finally in 1 % uranyl acetate solution. Afterwards, the sections were dehydrated, embedded in Durcupan resin (Fluka), mounted between two microscope slides and then hardened for 48 h at 60°C.

Regions of interest were selected using bright-field microscopy, and then cut using a razor blade. Subsequent section processing included gluing to a resin block, trimming and further sectioning. Sections of 50 nm thickness were collected onto slot grids with a formvar support film. Images were taken at 80 kV using a transmission electron microscope (Tecnai Spirit FEI).
