# Supplementary material for: Modulating the catalytic activity of AMPK has neuroprotective effects against α-synuclein toxicity
Source: Mol Neurodegener. 2017 Nov 3;12:80. doi: 10.1186/s13024-017-0220-x (PMC5670705; doi:10.1186/s13024-017-0220-x)
Supplement: Supplementary file 2 — Figure S1. Levels of phospho-ACC in HEK293T cells overexpressing AMPKα. (PDF 352 kb) [file 13024_2017_220_MOESM2_ESM.pdf]

Figure S1

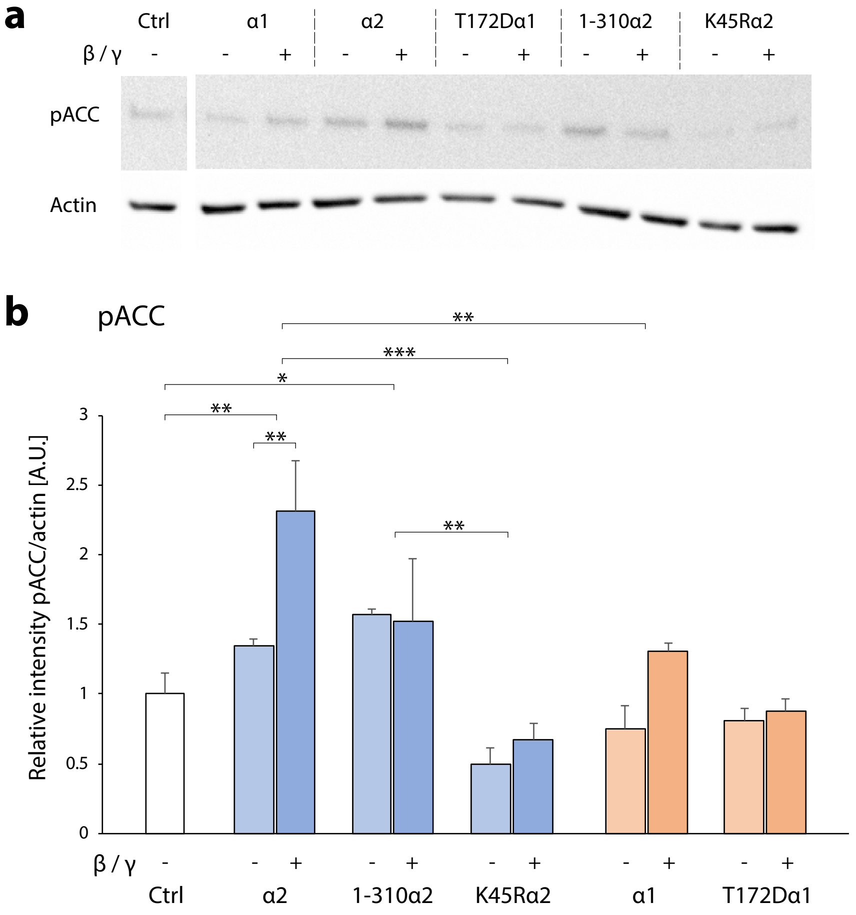

Fig S1. Levels of phospho-ACC in HEK293T cells overexpressing AMPKα

(**a**) Representative immunoblotting of protein extracts, 48 hrs after transfection of KEK293T cells with each of the various pAAV-AMPKα constructs, either alone, or co-transfected with plasmids expressing the β1 and γ3 subunits of the AMPK complex. Immunodetection of phospho-ACC (pACC) and actin.
(**b**) Quantification of relative pACC levels analyzed by western blotting. The pACC signal is normalized to actin. The pAAC level is set at 1 in HEK293T transfected with the control non-coding pAAV plasmid (Ctrl). Note that only the wild-type α1 and α2 subunits change the level of pACC as a function of β1/γ3 subunit expression. The activity of the other subunits appears independent from the β and γ AMPK subunits. Note the significant reduction of the pACC signal in neurons overexpressing the dominant-negative K45Rα2, compared to α2 and 1-310α2 variants.

Values are expressed as mean±SEM. Statistical analysis: two-way factorial ANOVA (AMPKα x β/γ co-expression) with Fisher's LSD post hoc test; n=2 per condition; *P<0.05, **P<0.01, ***P<0.001.
