# Supplementary material for: Modulating the catalytic activity of AMPK has neuroprotective effects against α-synuclein toxicity
Source: Mol Neurodegener. 2017 Nov 3;12:80. doi: 10.1186/s13024-017-0220-x (PMC5670705; doi:10.1186/s13024-017-0220-x)
Supplement: Supplementary file 3 — Figure S2. Levels of AMPKα1 and α2 transcripts in primary mouse cortical neurons and in the rat SN. (PDF 855 kb) [file 13024_2017_220_MOESM3_ESM.pdf]

Figure S2

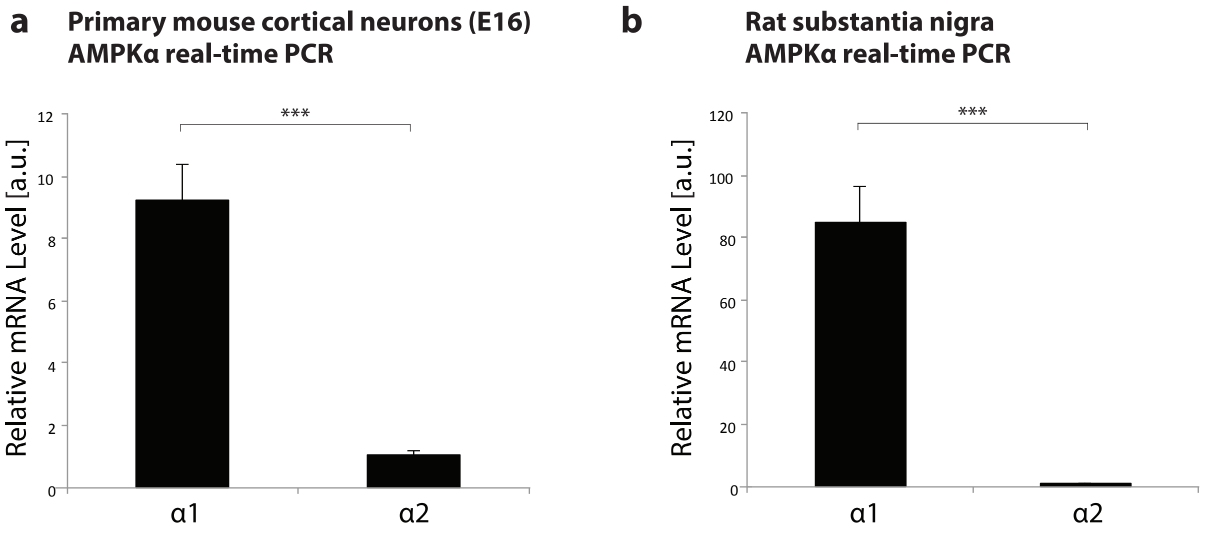

Fig S2. Levels of AMPKα1 and α2 transcripts in primary mouse cortical neurons and in the rat SN

(**a**) Real-time PCR quantification of endogenous mRNA levels of AMPKα1 and α2 subunits in primary mouse cortical neurons.

(**b**) Real-time PCR quantification of endogenous mRNA levels of AMPKα1 and α2 subunits in rat SN. Note in both cases the significantly higher transcript level for the AMPKα1 subunit.

Relative values of gene expression are expressed as mean±SEM, calculated for each individual sample using the ΔΔCt method; (a) n=6 separate wells of neuronal primary cultures; (b) n=6 separate tissue samples from the SN, each originating from a different animal; Student's t-test: ***P<0.001.
