# Supplementary material for: Modulating the catalytic activity of AMPK has neuroprotective effects against α-synuclein toxicity
Source: Mol Neurodegener. 2017 Nov 3;12:80. doi: 10.1186/s13024-017-0220-x (PMC5670705; doi:10.1186/s13024-017-0220-x)
Supplement: Supplementary file 4 — Figure S3. Levels of phospho ACC in primary cortical neurons overexpressing AMPKα and α-syn. (PDF 222 kb) [file 13024_2017_220_MOESM4_ESM.pdf]

Figure S3

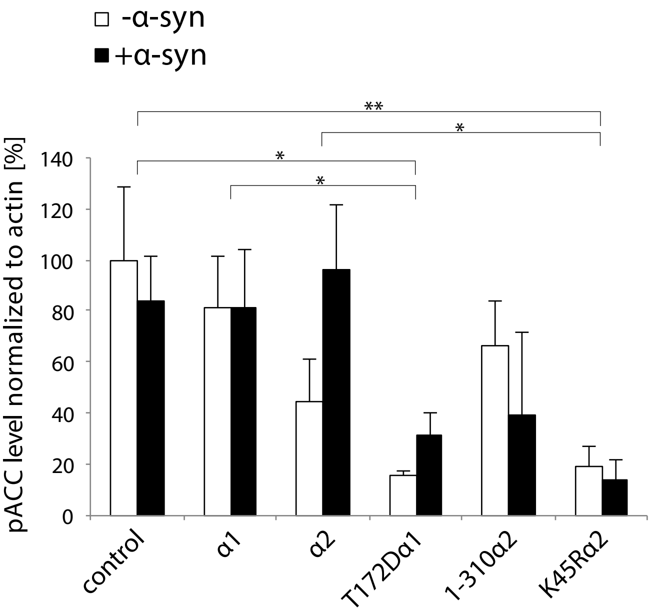

Fig S3. Levels of phospho ACC in primary cortical neurons overexpressing AMPKα and α-syn

Quantification of relative phospho ACC (pACC) levels analysed by western blotting (see Fig 1b for original blot). The pACC signal is normalized to actin. The pAAC level is set at 100% in primary neurons transduced with the control non-coding AAV6 vector. Note the significant reduction of the pACC signal in neurons overexpressing AMPK T172Dα1 and K45Rα2.

Values are expressed as mean±SEM. *Statistical analysis*: repeated measures two-way ANOVA with Fisher's LSD post hoc test; n=2 per condition; *P<0.05, **P<0.01.
