# Supplementary material for: Modulating the catalytic activity of AMPK has neuroprotective effects against α-synuclein toxicity
Source: Mol Neurodegener. 2017 Nov 3;12:80. doi: 10.1186/s13024-017-0220-x (PMC5670705; doi:10.1186/s13024-017-0220-x)
Supplement: Supplementary file 5 — Figure S4. Down-regulation of dopaminergic markers in the nigrostriatal system following overexpression of human α-syn and T172Dα1 AMPK subunit. (PDF 1785 kb) [file 13024_2017_220_MOESM5_ESM.pdf]

Figure S4

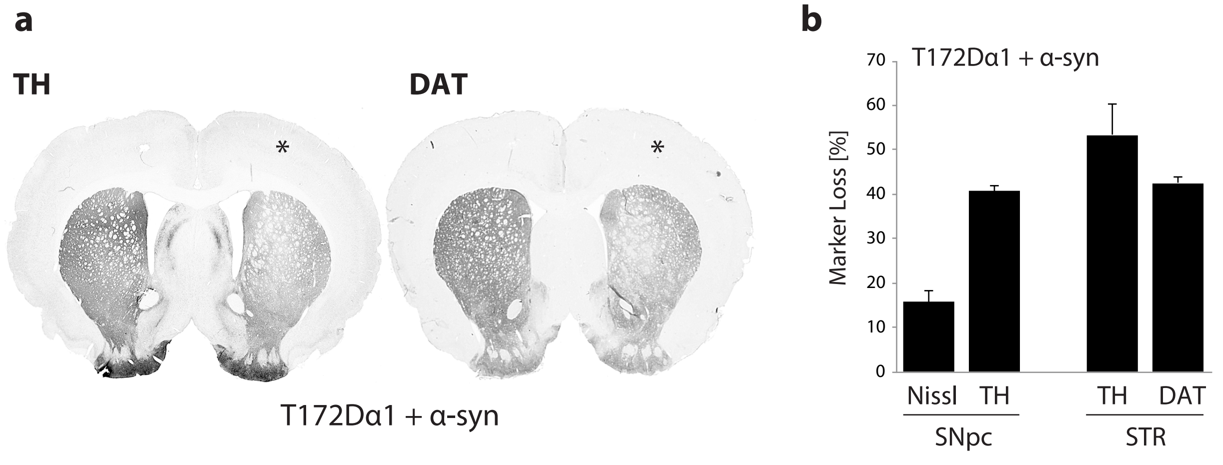

Fig S4. Down-regulation of dopaminergic markers in the nigrostriatal system following overexpression of human α-syn and T172Dα1 AMPK subunit

(**a**) Representative immunostaining for TH and DAT in the STR of an animal co-injected with AAV-α-syn and AAV-T172Dα1.

(**b**) Comparison of the α-syn-induced loss of nigral neurons and striatal fiber densitometry, using Nissl as well as the DAT and TH dopaminergic markers in rats co-injected with AAV-α-syn and AAV-T172Dα1 (n=5 for each marker). Note the higher loss of dopamine marker immunoreactivity with respect to the loss of Nissl-stained neurons in the SNpc.

* indicates the injected hemisphere.
