# Supplementary material for: Modulating the catalytic activity of AMPK has neuroprotective effects against α-synuclein toxicity
Source: Mol Neurodegener. 2017 Nov 3;12:80. doi: 10.1186/s13024-017-0220-x (PMC5670705; doi:10.1186/s13024-017-0220-x)
Supplement: Supplementary file 6 — Figure S5. Number of mCherry+/GFP+ autophagosomes in primary cortical neurons overexpressing AMPKα and/or α-syn. (PDF 221 kb) [file 13024_2017_220_MOESM6_ESM.pdf]

Figure S5

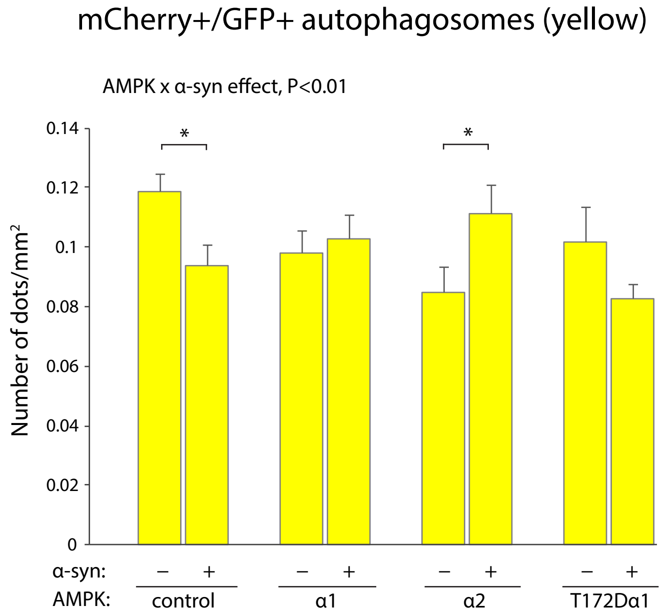

Fig S5. Number of mCherry+/GFP+ autophagosomes in primary cortical neurons overexpressing AMPKα and/or α-syn

Quantification of the average number of EGFP-positive autophagosomes per cell, normalized to cytosol area. Note that in neurons overexpressing α-syn, the number of autophagosomes remains similar across the different conditions, regardless of AMPKα overexpression.

Values are expressed as mean±SEM. *Statistical analysis*: two-way factorial ANOVA with Fisher's LSD post hoc test; for each condition n=22-30 neurons from 3 separately infected wells; *P<0.05.
