# Supplementary material for: Modulating the catalytic activity of AMPK has neuroprotective effects against α-synuclein toxicity
Source: Mol Neurodegener. 2017 Nov 3;12:80. doi: 10.1186/s13024-017-0220-x (PMC5670705; doi:10.1186/s13024-017-0220-x)
Supplement: Supplementary file 7 — Figure S6. Mitochondrial respiration in cortical neurons co-expressing AMPKα and α-syn. (PDF 144 kb) [file 13024_2017_220_MOESM7_ESM.pdf]

Figure S6

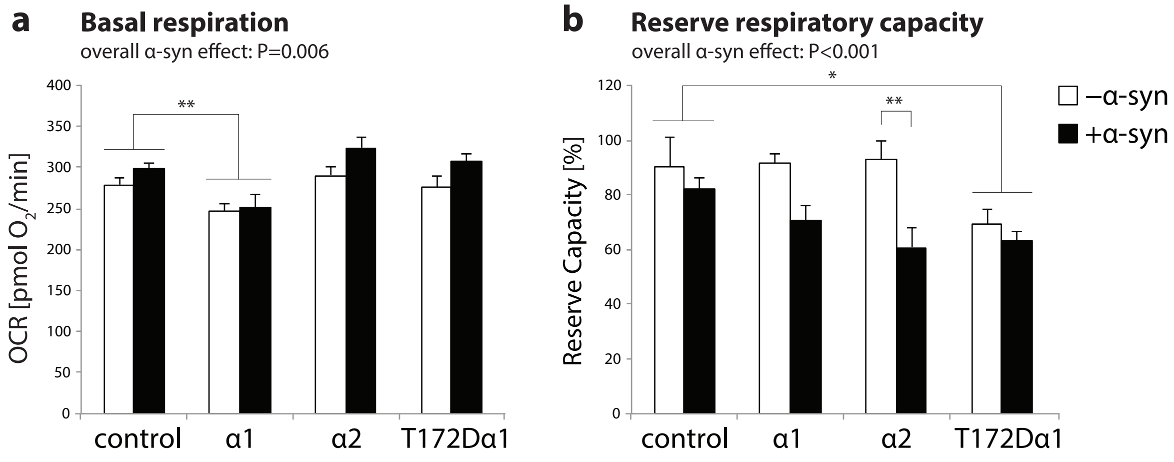

Fig S6. Mitochondrial respiration in cortical neurons co-expressing AMPKα and α-syn

(**a**) Basal oxygen consumption rate (OCR), expressed as the mean of four measurements. Note that AMPKα1 overexpressing neurons have statistically lower basal OCR compared to control, whilst α-syn induces an overall increase in OCR. Significant differences in the multiple comparisons are indicated on the graph for the AMPK and α-syn effects.

(**b**) Reserve respiratory capacity of neurons across conditions, calculated as the percentage increase in OCR following CCCP exposure. Overexpression of α-syn results in a significant decrease in the reserve respiratory capacity, in particular in neurons overexpressing AMPKα1 and α2. As compared to the control condition, neurons overexpressing T172Dα1 show a lower reserve respiratory capacity, independently from α-syn overexpression. Significant differences in the multiple comparisons are indicated on the graph for the AMPK and α-syn effects.

Statistical analysis: two-way factorial ANOVA (α-syn x AMPKα) with Tukey's HSD post hoc test; n=10-12 wells of separately infected neurons; *P<0.05, **P<0.01.
